# Metal Solidification Imaging Process by Magnetic Induction Tomography

**DOI:** 10.1038/s41598-017-15131-z

**Published:** 2017-11-06

**Authors:** Lu Ma, Stefano Spagnul, Manuchehr Soleimani

**Affiliations:** 1 University of Bath, Engineering Tomography Laboratory, Department of Electronic and Electrical Engineering, Bath, BA2 7AY UK; 2 Ergolines lab s.r.l., Area Science Park, Bldg. R3 Padriciano, Trieste, 34149 Italy

## Abstract

There are growing number of important applications that require a contactless method for monitoring an object surrounded inside a metallic enclosure. Imaging metal solidification is a great example for which there is no real time monitoring technique at present. This paper introduces a technique - magnetic induction tomography - for the real time *in-situ* imaging of the metal solidification process. Rigorous experimental verifications are presented. Firstly, a single inductive coil is placed on the top of a melting wood alloy to examine the changes of its inductance during solidification process. Secondly, an array of magnetic induction coils are designed to investigate the feasibility of a tomographic approach, i.e., when one coil is driven by an alternating current as a transmitter and a vector of phase changes are measured from the remaining of the coils as receivers. Phase changes are observed when the wood alloy state changes from liquid to solid. Thirdly, a series of static cold phantoms are created to represent various liquid/solid interfaces to verify the system performance. Finally, a powerful temporal reconstruction method is applied to realise real time *in-situ* visualisation of the solidification and the measurement of solidified shell thickness, a first report of its kind.

## Introduction

There are numerous applications require seeing through metal, such as combating illicit trafficking of special nuclear material within metallic cargo containers^1^. Monitoring the molten metal solidification process in the steel manufacturing industry is also a great example^2^. In this process, molten steel is poured into a tundish and then into a water-cooled copper mould through a device at the bottom of tundish to allow the flow of steel into the mould. Within the mould, the outer shell of the steel becomes solidified to form a steel strand with the liquid steel inside. The solidifying strand is continuously withdrawn from the mould into additional cooling chambers to promote solidification. Once the strand is fully solidified, it is cut to the steel slabs in various lengths to meet downstream product specifications. Therefore it is important to visualise the *in-situ* solidification process and measure the solidified shell thickness continuously in a non-intrusive, non-contact and real time manner for process and quality control.

Magnetic induction tomography (MIT) relies on eddy currents induced voltages from receiving coils in a time-varying magnetic field to inversely image a conductive subject. Theoretical and simulation work have previously looked at the imaging of solidification of molten metal inside a pipe by eddy currents from a metallurgy and a computational electromagnetic point of view^3,4^. Studies have also been done to explore the feasibility and requirements of using MIT for the imaging of molten flow within the pouring nozzle between the tundish and the mould. Different flow regime models were created using woods metal and glass beads to present argon gas bubbles to facilitate the system design specification^5^. MIT imaging results from a 20 seconds hot trial were reported and demonstrated that the reconstructed images of motel steel flow have a good consistency with video recordings from an exposed section of the steel flow in terms of flow size and position^6^. In addition, an MIT system was used to visualise the behavior of two-phase GaInSn/argon flow through the submerged entry nozzle (made of acrylic glass) during continuous casting and demonstrated that it is feasible to distinguish different flow regimes under various argon flow rate and stopper rod positions^7,8^.

The contributions of this study are threefold. Firstly, the subject of study is not the metal flow within the entry nozzle between the tundish and the mould or how the flow regimes change as have been studied previously, instead this paper studies and visualises the metal solidification process within the mould itself. Secondly, this work provides a full tomographic approach for the real time *in-situ* monitoring of the solidification through a metallic shell, making the realisation of the technique viable in an industrial environment. Thirdly, the solidified shell thickness within the mould are estimated using temporal correlated images, an important process parameter for industrial users. Taken together, this is believed to be the first study of its kind in the field of metal solidification imaging.

## Solidification measurement

**Figure 1:**
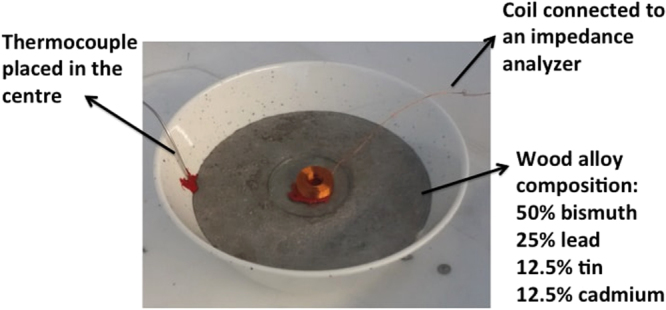
Experimental setup of a single coil placed on the top of a melting wood alloy.

A MIT system uses an array of inductive coils to measure the induced voltages from eddy currents effect in a time varying magnetic field. For metal solidification process, it is essential to understand the behaviour of the coil measurement arising from the metal state change from liquid to solid phase. An experiment is performed using a single coil placed on the top of a low melting wood alloy to study the change of the coil measurement for proof of principles (Fig. 1). The composition of the wood alloy is 50% Bismuth, 25% Lead, 12.5% Tin and 12.5% Cadmium. The electrical conductivity $\sigma$ is 1.9 MS/s and the relative permeability ${\mu }_{r}$ is 2. The wood alloy is an eutectic alloy, with a density of 9.6 $g/c{m}^{3}$ and a melting point of 72 °C, which can be easily reached using hot water. The coil is made of copper wire with a diameter of 0.25 mm. The inner and outer radii are 6 and 10 mm respectively. This is a multi-layer (26 layers in total), multi-turn (17 turns per layer) air-cored inductor with an inductance of 2.5 mH measured in a free space and a DC resistance of 8.2 ohm. The coil is driven at a frequency of 130 Hz. The standard penetration depth can be defined as: $\delta =\sqrt{2/\omega {\mu }_{o}{\mu }_{r}\sigma }$, where ${\mu }_{0}$ is the free space permeability. At 130 Hz, the penetration depth is 22.6 mm through the wood alloy. The electrical impedance of the coil and temperature of the wood alloy during the solidification process are recorded using a HIOKI 3522-50 impedance analyzer.

**Figure 2:**
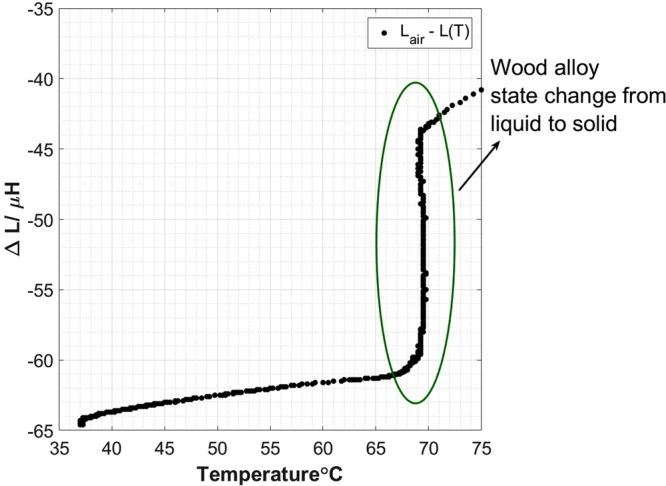
Measurements of the coil inductance differences versus the temperature changes during the solidification of a wood alloy.

For an eutectic alloy, theoretically there is no mushy zone and the change of state from liquid to solid occurs within a couple of degrees. Figure 2 shows the measured coil inductance difference with respect to air versus temperature during the solidification of the wood alloy. A rapid change in the inductance difference is observed between temperature 68–70 °C, corresponding to the physical metal state change.

**Figure 3:**
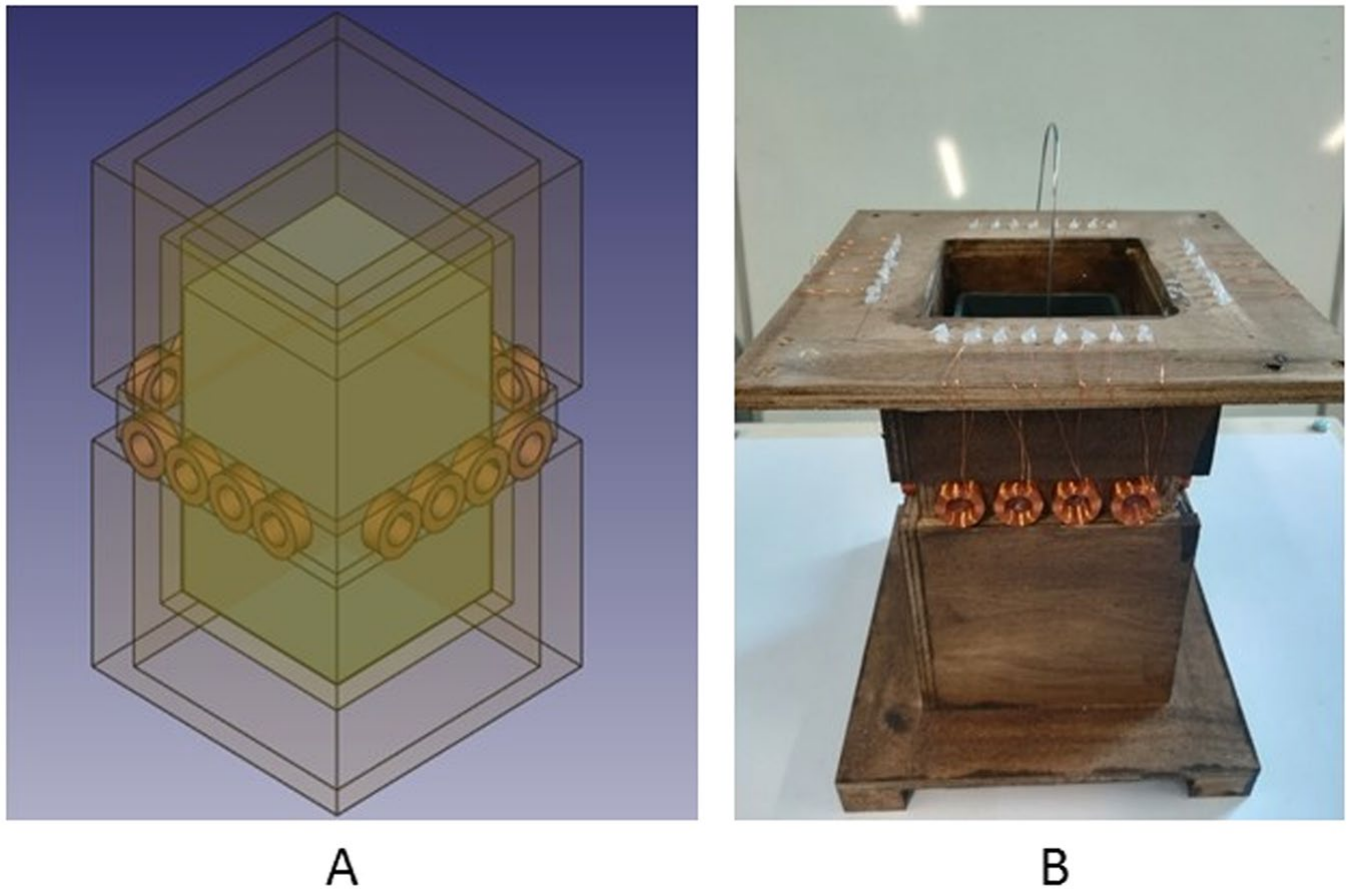
A schematic design of the MIT prototype design (**A**) and a true picture (**B**) of the experimental setup to measure the phase changes during the solidification of a wood alloy.

**Figure 4:**
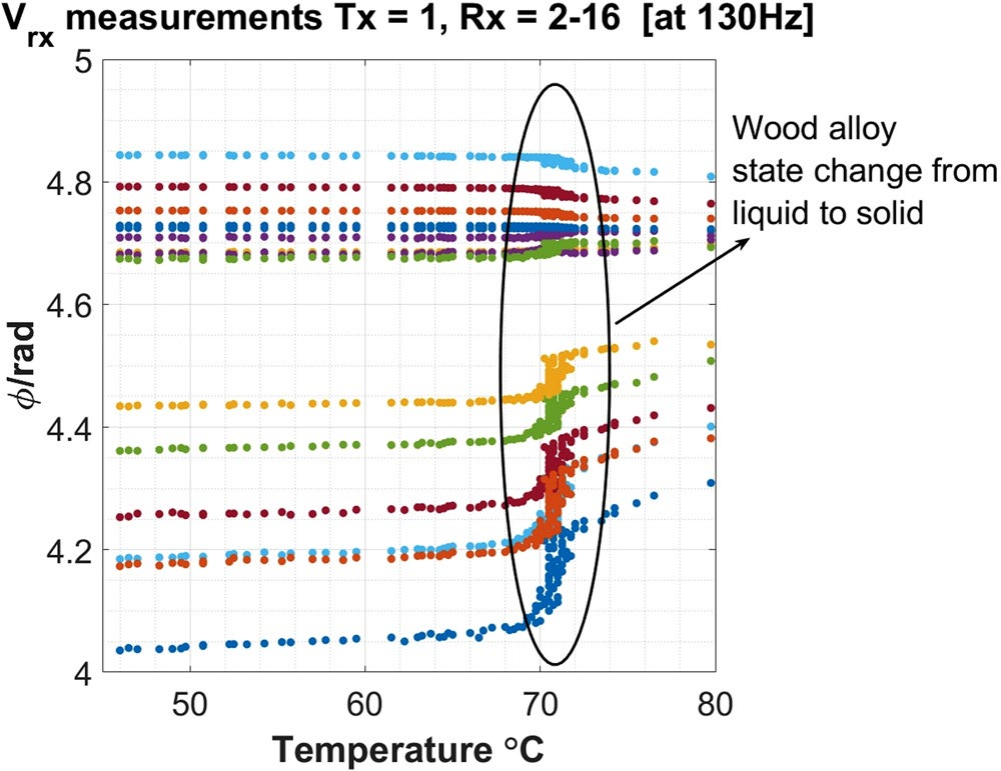
Phase measurements from all the receiving coils versus the temperature changes during the solidification of a wood alloy.

Based on Fig. 2, a MIT prototype is designed to investigate the induced voltages from an array of measurement coils, which are essential for image reconstruction (Fig. 3). As the imaging subject in a solidification process usually takes in the shape of a billet in a square mould, a 4-by-4 square coil array is design to accommodate this geometry with a distance of 98 mm between two opposite plane of coils. Each coil has the same geometrical and electrical properties as the one used in the single coil test (Fig. 1). A plastic mould container with inner dimension of 74 mm (length) -by-74 mm (width) -by-120 mm (depth) is positioned in the centre of the coil array. Approximately 6 Kg of wood alloy is melted using hot water and poured into the plastic mould. A thin k-type thermocouple is held in the centre of the block and the temperatures are continuously recorded. Figure 4 shows the phase measurements from all the receiving coils number 2 to 16 when transmitting coil number 1 is driven by a 300 mA current at 130 Hz. Similarly, clear changes in the phase measurements are observed when the metal changes from a liquid to a fully solidified state, consistently with Fig. 2.

## Hardware

**Figure 5:**
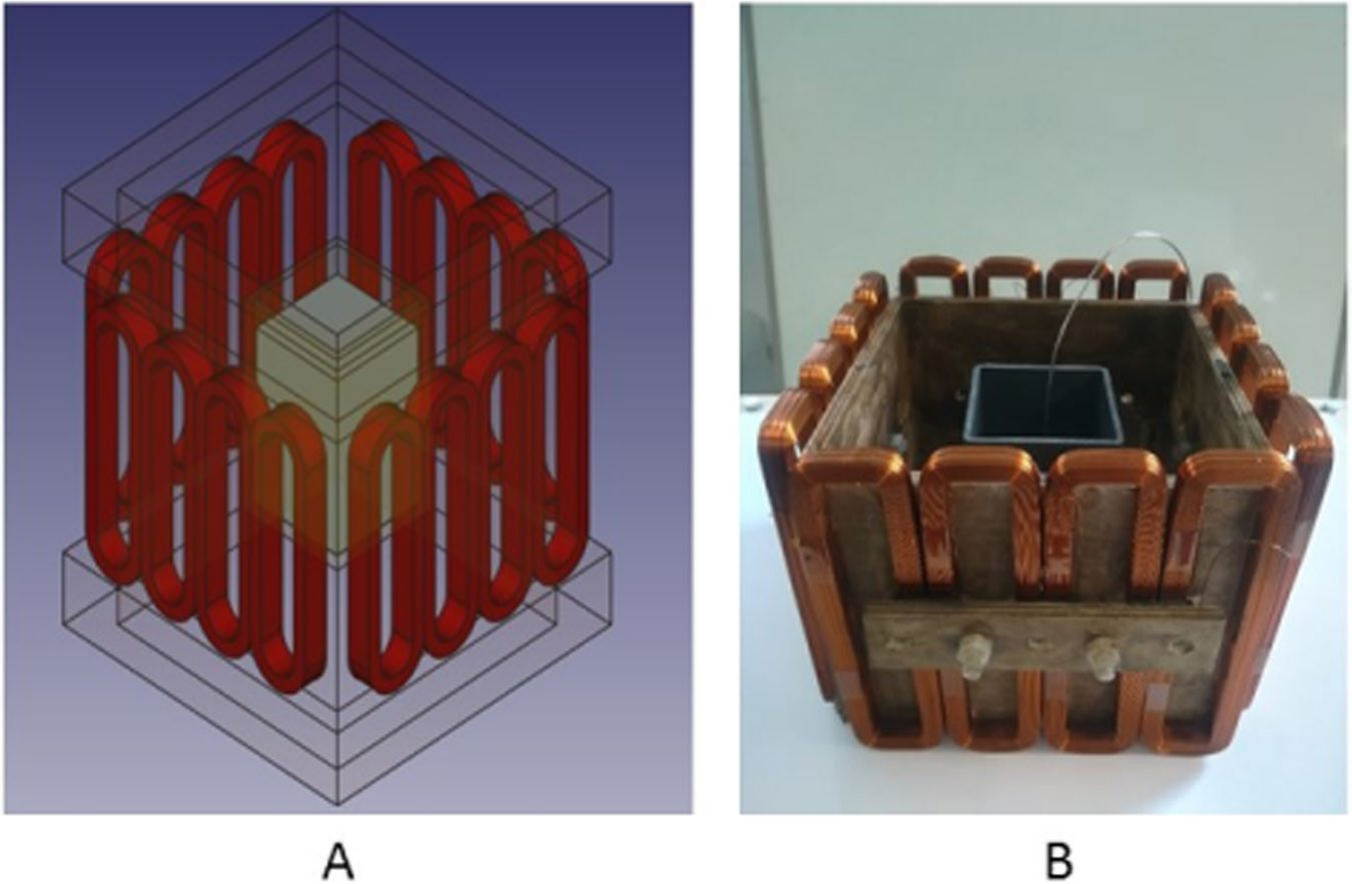
A schematic design (**A**) and a true picture of the MIT coil array (**B**) proposed for metal solidification imaging.

The MIT prototype system demonstrates the feasibility of distinguishing the metal state change from its phase measurements from the receiving coils. From the experimental trials, it was discovered that a major drawback of this prototype is that the induced voltage signals from opposite coils are too small (a hundred of microvolts), posing a high demand on the amplification of the signals and consequently reducing the signal-to-noise ratio (SNR) of the system. In addition, increasing the radius of the coils is not a viable option though larger coils would have higher magnetic field strength. This is because in order to maintain a 4-by-4 square coil array geometry, increasing the coil radius results in an further increase in the distance between opposite coils. To overcome this dilemma, a rectangular coil is proposed with the view to gain up to several orders of magnitude in the induced voltages from opposite coils without extremely high requirement of the amplification. In the final MIT system (Fig. 5), each coil remains a multi-layer, multi-turn air-cored inductor, however the coil shape changed from circular to rectangular. Each coil has 20 layers and 20 turns per layer. The coil wire diameter is 0.5 mm. The inner and outer widths of each coil are 27 and 47 mm, the inner and outer lengths of the coil are 162 and 182 mm respectively. The coil thickness is 11.4 mm. The coil inductance measured in air is 23.66 mH and the DC resistance is 14.69 ohm.

**Figure 6:**
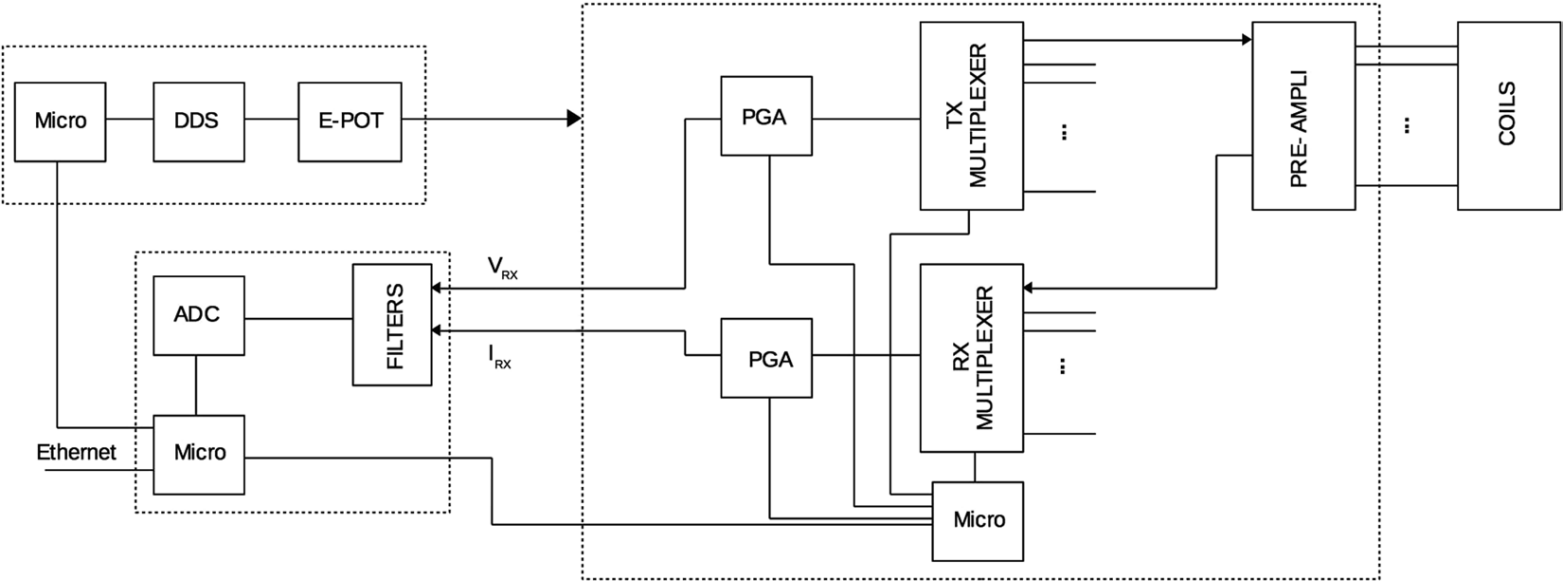
A schematic design of the hardware of the MIT system proposed for metal solidification imaging.

The coil array is connected to an electronic system, comprises a front-end electronics component, a signal acquisition card and a signal generation unit (Fig. 6). The signal generator is a digital potentiometer driven by a Direct Digital Synthesizer (DDS) AD9832 operating at 25 MHz, with a frequency accuracy can be controlled to one part in 4 billion. The potentiometer is a nonvolatile digital potentiometer that has 100 positions. The front-end electronics component contains a multiplexer, which drives both the transmitting and receiving coils. A pre-amplification for each coil is located immediately after the coils’ connectors using an instrumentation amplifier INA128. The signal is amplified by a PGA downstream of the multiplexer. The maximum gain is 8000, while the bandwidth is 1 MHz. The system drives a maximum current of 500 mA with a maximum frequency of 4000 Hz and a maximum voltage of ±10 V. The induced voltages are sampled by a 16 bit 250 kSPS ADC and their amplitudes and phases can be extracted using a Fast Fourier Transform method, after a Sallen-Key low pass butterworth filter. The communication is based on User Datagram Protocol (UDP) via a Microchip controller ENC424J600 (Micro). The overall SNR of the system is over 80 dB. At 130 Hz, the acquisition rate for a complete scan is approximately 80 seconds, sufficient to detect the overall changes in electrical conductivity during the solidification of a wood alloy.

## Methods

MIT utilises an array of inductive coils, distributed equally around an imaging region, to visualise the electromagnetic property distribution of the conductivity of an imaging subject. The imaging principle is based on the laws of induction and eddy currents which are induced in an AC magnetic field. The governing equation can be written as equation 1:

1 $$\nabla \times \frac{1}{\mu }\nabla \times A+j\omega \sigma A={J}_{s}$$

where $\mu$ is the permeability, $A$ is the total magnetic vector potential as a result of the current source ${J}_{s}$ and the effect of eddy current induced by the the conductivity $\sigma$, and $\omega$ is the angular frequency. The current density ${J}_{s}$ can be prescribed by the magnetic vector potential according to the Biot-Savart Law. Equation 1 is solved by approximating the system as a combination of linear equations in small elements with appropriate boundary conditions using the Galerkin’ s approximation^9^, where $S$ is the complex system matrix, and $b$ is the right hand side current density. The eddy current region magnetic vector potential ${A}_{e}$ can be solved and used to formulate the Jacobian (sensitivity) matrix^10^.

2 $$S{A}_{e}=b$$

The inversion of MIT data is an ill-posed inverse problem. As the inverse problem involves inverting the sensitivity matrix, which makes the solution unreliable and sensitive to modelling error and measurement noise. Consequently, a small measurement or modelling error could results in a very large change in the reconstructed conductivity profile. Implementing regularisation techniques that involve introducing additional penalty terms and a smoothing parameter usually mitigates this problem. The inverse problem can usually be formulated in terms of optimising an object function with physical measurements and the goal is to solve the distribution of conductivity (in the context of this study), while the measurement signals are given. Solving the inverse problem includes starting with a trial configuration of the system parameters and subsequently modifying this configuration using iterative or non-iterative optimisation algorithms. A one-step Tikhonov based hybrid image reconstruction algorithm is used to reconstruct images for cold tests:

3 $${\rm{\Delta }}\sigma ={({J}^{T}J+\alpha {R}_{1}+\beta {R}_{2}+\gamma {R}_{3})}^{-1}{J}^{T}{\rm{\Delta }}{\varphi }$$

where ${\rm{\Delta }}{\varphi }$ is the measured phase different with respect to a reference phase measurement. $J$ is the sensitivity matrix computed from the forward model. ${\rm{\Delta }}\sigma$ is the target conductivity. There are three penalty terms used to find the stable solution: ${R}_{1}$ is diagonal component of the ${J}^{T}J$, ${R}_{2}$ is an identity matrix and ${R}_{3}$ is a Laplacian operator (a weight matrix) based on the number of grids in the reconstruction to smooth the image. Their corresponding regularisation parameters are $\alpha$, $\beta$ and $\gamma$.

For the wood alloy solidification imaging, a temporal solution is is adopted to take the advantage of the temporal resolution from the phase measurements between time frames^11^. The time period for the solidification process is $T$ from ${t}_{-d}$ to ${t}_{d}$, within this period, a series of phase difference measurements are captured: $\tilde{{\rm{\Delta }}\varphi }=[{\rm{\Delta }}{{\varphi }}_{-d},{\rm{\Delta }}{{\varphi }}_{-d+\mathrm{1,}},\mathrm{...,}\,{\rm{\Delta }}{{\varphi }}_{d-1},{\rm{\Delta }}{{\varphi }}_{d}]$, and the corresponding reconstructed conductivity changes are $\tilde{{\rm{\Delta }}\sigma }=[{\rm{\Delta }}{\sigma }_{-d},{\rm{\Delta }}{\sigma }_{-d+\mathrm{1,}},\mathrm{...,}\,{\rm{\Delta }}{\sigma }_{d-1},{\rm{\Delta }}{\sigma }_{d}]$. The concatenated Jacobian matrix $\mathop{J}\limits^{\sim }=I\otimes J$, where the identity matrix $I$ has a size of $2d+1$. The $\otimes$ notation is the Kronecker tensor product, which results in $\mathop{J}\limits^{\sim }$ as a block diagonal matrix. Using this approach, equation 3 can be re-written as:

4 $$\tilde{{\rm{\Delta }}\sigma }=(\mathop{P}\limits^{\sim }\mathop{{J}^{T}}\limits^{\sim })(\mathop{J}\limits^{\sim }\mathop{P}\limits^{\sim }\mathop{{J}^{T}}\limits^{\sim }+\lambda \tilde{{W}^{-1}}{)}^{-1}\tilde{{\rm{\Delta }}{\varphi }}$$

where $\mathop{W}\limits^{\sim }=I\otimes W$, $W$ is the regularisation matrix for the measurement noise and $\lambda$ is the regularisation parameter. $\mathop{{P}}\limits^{\sim }=T\otimes P$ where $T$ is the regularisation matrix resulting from $\alpha {R}_{1}+\beta {R}_{2}+\gamma {R}_{3}$ that represents the temporal correlation between the sequential images. In this study, two approximations are made: firstly the noise between frames of data are considered independent; secondly the Jacobian matrix is considered constant, though equation 4 can also be formulated to take into consideration of the time varying Jacobian matrix.

## Cold tests

**Figure 7:**
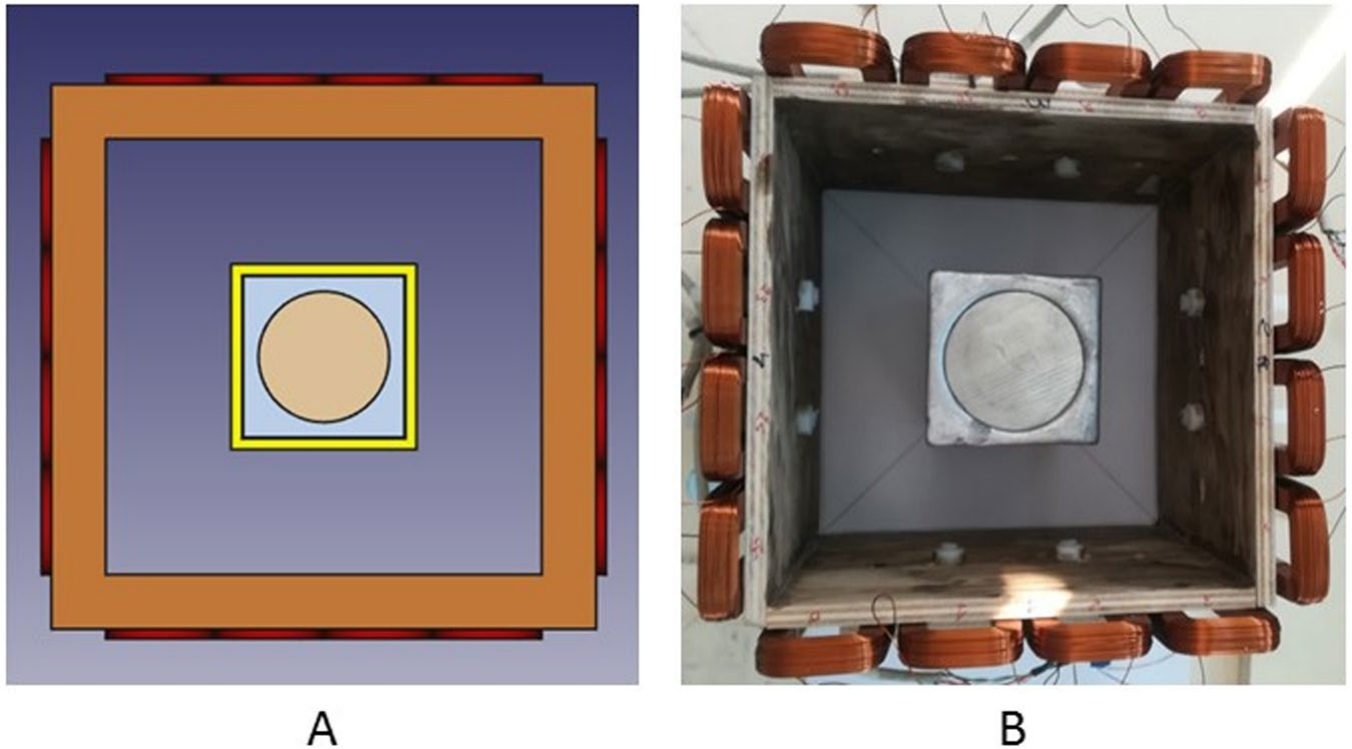
A schematic representation (**A**) and a true experimental setup (**B**) of the cold phantom tests, the central rod diameters vary from 60, 50, 35, 32 to 30mm in each test.

To verify the performance of the proposed MIT system, some cold phantom tests are designed as analogue of various stages of the solidification process (Fig. 7). A square billet with a dimension of 74 mm (length) -by-74 mm (width) -by-120 mm (depth) is used to represent the liquid metal at the beginning of the solidification process. The square billet is made of wood metal with the same composition and electrical properties stated in previous section. In the centre of the billet, rods with diameters of 60, 50, 35, 32 and 30 mm are cast to represent the liquid zone during the solidification process. The phantoms are created in such a way that there is no air gap between the square metal shell and the central rod. The rods are made of stainless steel aisi303, which has a conductivity of 1.4 MS/m and a relative permeability of 1.

**Figure 8:**
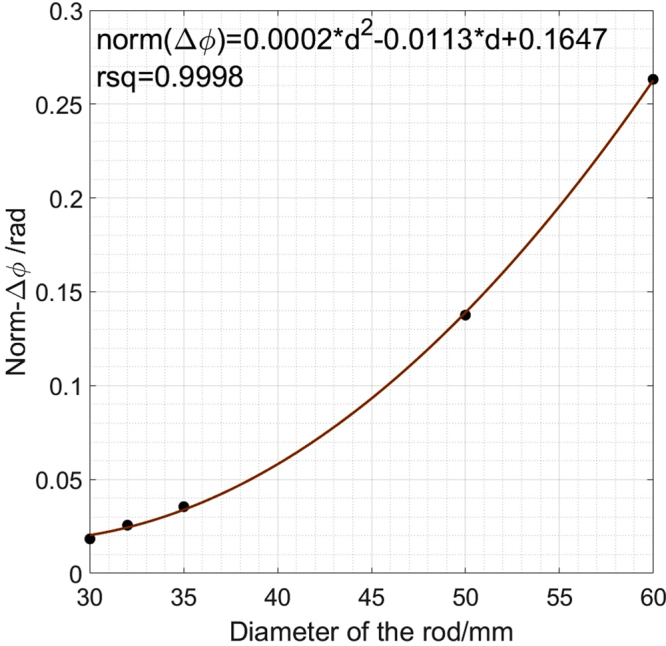
Relationship between the norm of measured phase differences versus the diameters of the rods.

**Figure 9:**
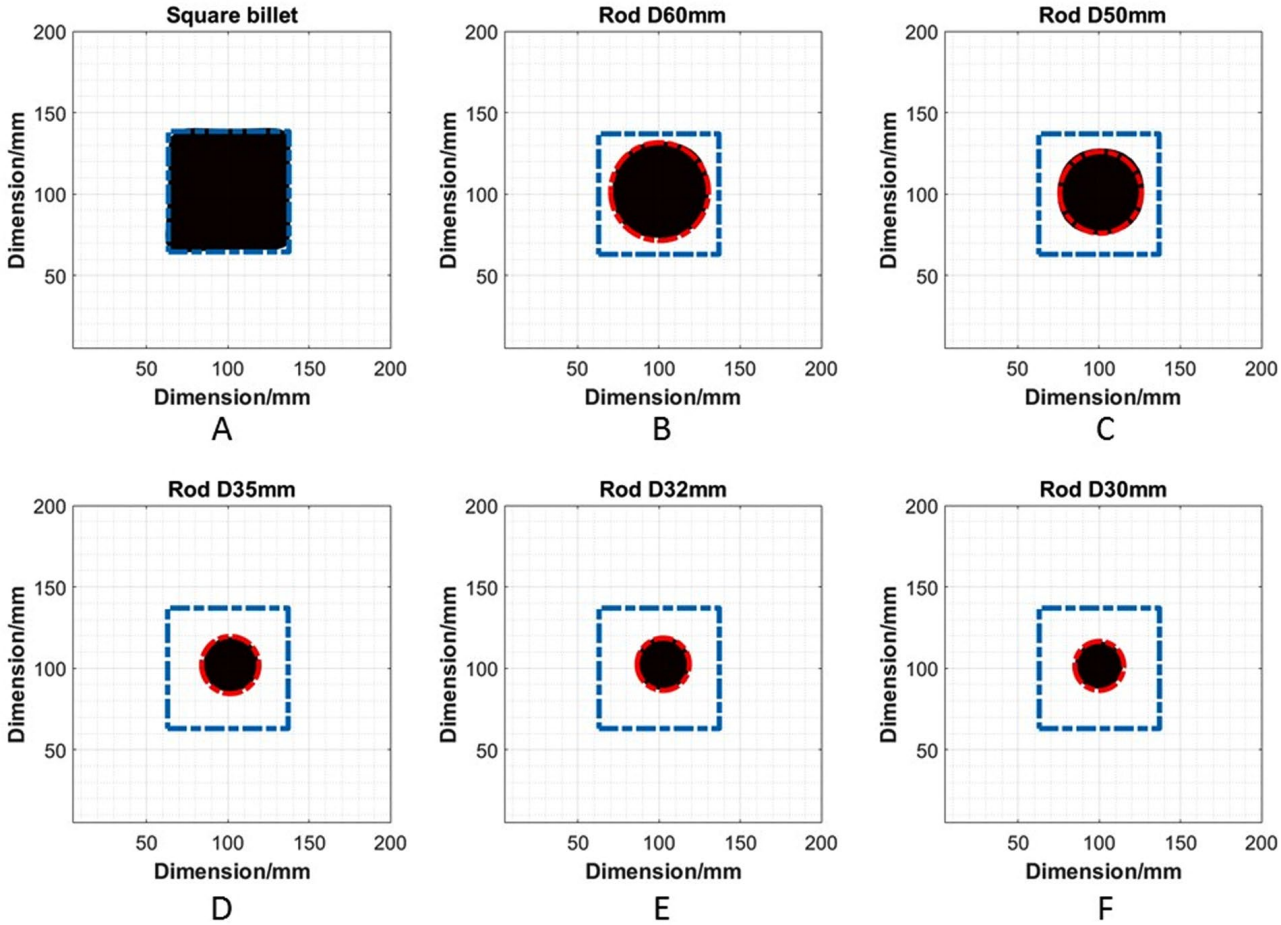
Reconstructed images of internal rods within metallic shells, a reconstructed square billet (**A**) to represent a liquid filled mould at the beginning of the solidification; reconstructed images of rods from (**B**–**F**) represent the liquid zone within a solidified metal shell with its diameter decreases from 60, 50, 35, 32 to 30 mm during the solidification process.

The imaging region is divided into 40-by-40 pixels to cover the 200-by-200 mm imaging region. In these colds tests, the phase difference is measured with respect to air, a classic time difference imaging approach, i.e., ${\rm{\Delta }}{\varphi }={\varphi }-{{\varphi }}_{air}$. Figure 8 shows that the norm of the phase measurements has a quadratic relationship with the diameter of the internal rods, consistent with previous report^12^. The norm of the phase differences are calculated using $norm\,({\rm{\Delta }}{\varphi })=\sqrt{{\sum }_{m\mathrm{=1}}^{256}{({\rm{\Delta }}{{\varphi }}_{m})}^{2}}$ (16 coils results in a total of 256 measurements). The measured phase difference can be written as a function of the physical and electrical properties of the imaging target, i.e., ${\rm{\Delta }}{\varphi }=F(\sigma ,\mu ,f,D)$. In this case, only the diameter of the rod $D$ is the changing parameter. Figure 9 shows the reconstructed contour images of the liquid/solid phantoms from a fully liquid state. The dashed square and circle in Fig. 9B–F indicate where the solid shell and liquid region should be (calculated based on the true dimensions of the square billet and rods) with respective to the imaging region. It is important to note that in reality the electrical properties of the liquid metal varies with the temperature and the liquid region may also not be as symmetrical and uniform as the machined rods. These created phantoms are idealised scenarios to prove that by establishing the relationship between ${\rm{\Delta }}{\varphi }$ and $D$, the internal rods can be reconstructed through a conductive shell with appropriate threshold parameters chosen from the quadratic curve.

## Solidification imaging

**Figure 10:**
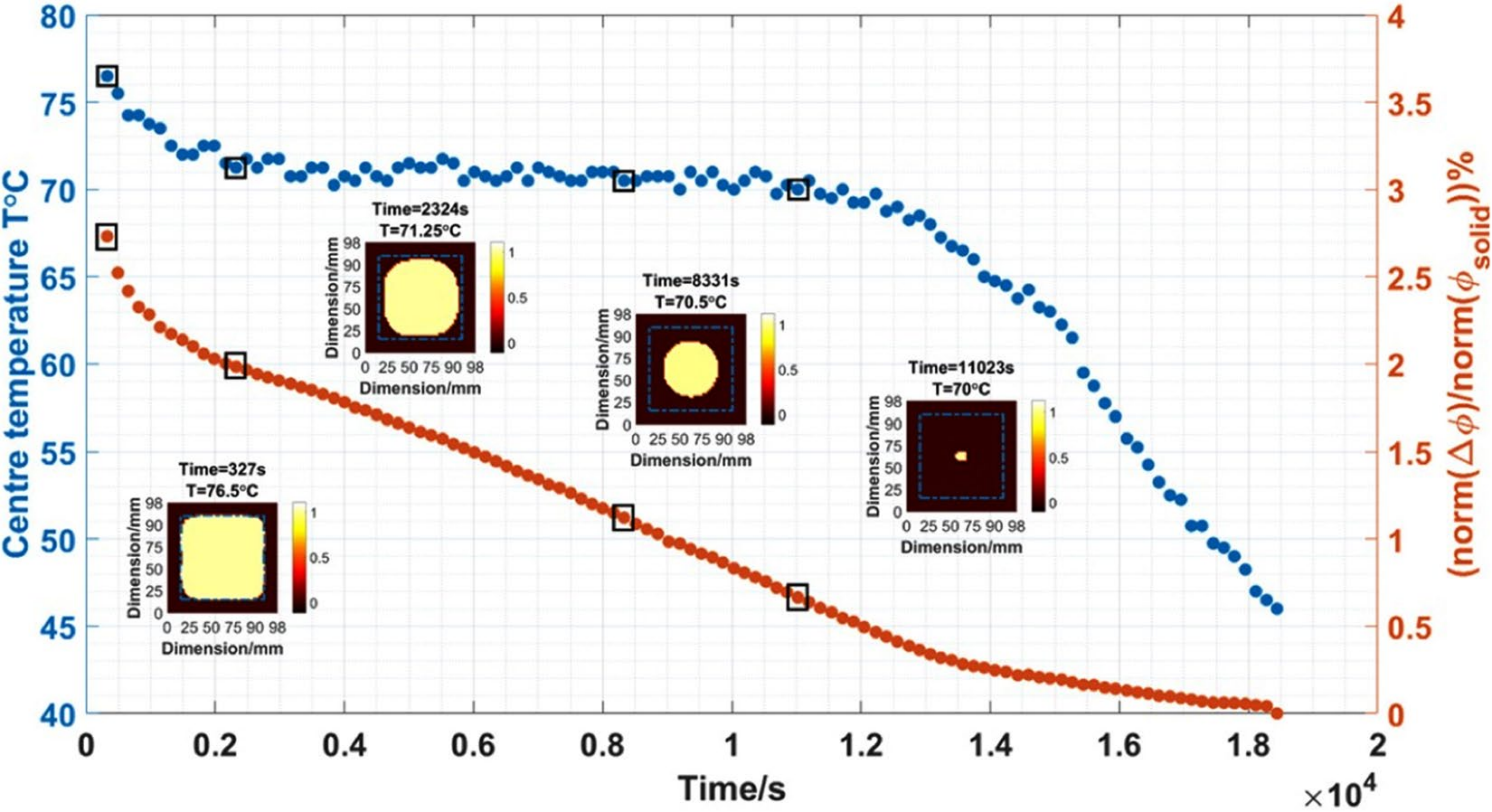
Phase measurement variation in percentage versus time and temperature during a solidification test using the MIT prototype (Fig. 3).

The experimental setup of solidification imaging of the wood alloy is similar to that of used in previous section. Wood alloy is melted using hot water and poured into a plastic mould positioned in the centre of the MIT system coil array. The reference phase measurements are taken from a fully solidified wood alloy, i.e., ${\rm{\Delta }}{\varphi }={\varphi }-{{\varphi }}_{solid}$. Figure 10 shows the phase measurement variation with respect to the reference measurement in percentage versus the temperature as well as time stamp. A selection of the temporal images are presented to demonstrate the status of the solidification process (data points highlighted in black squares). Noting that this experiment was performed using the MIT prototype system (Fig. 3), the imaging region is 98-by-98 mm. This experiment further verifies that the phase measurement changes in Fig. 4 are due to the metal state change and can be used to reconstructed images.

**Figure 11:**
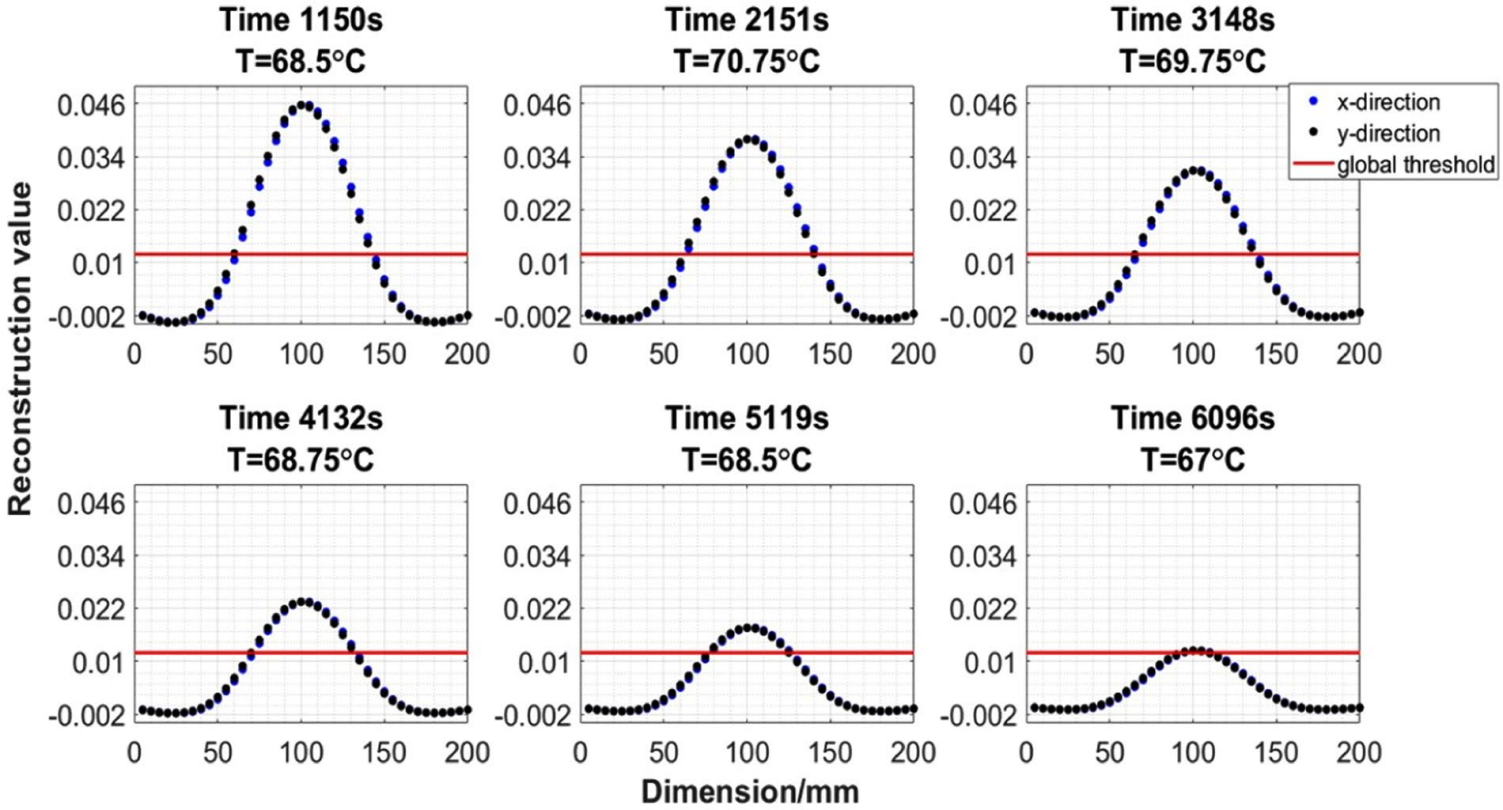
A selection of 1D distributions of the raw values from reconstructed images along both x- and y-axial directions.

**Figure 12:**
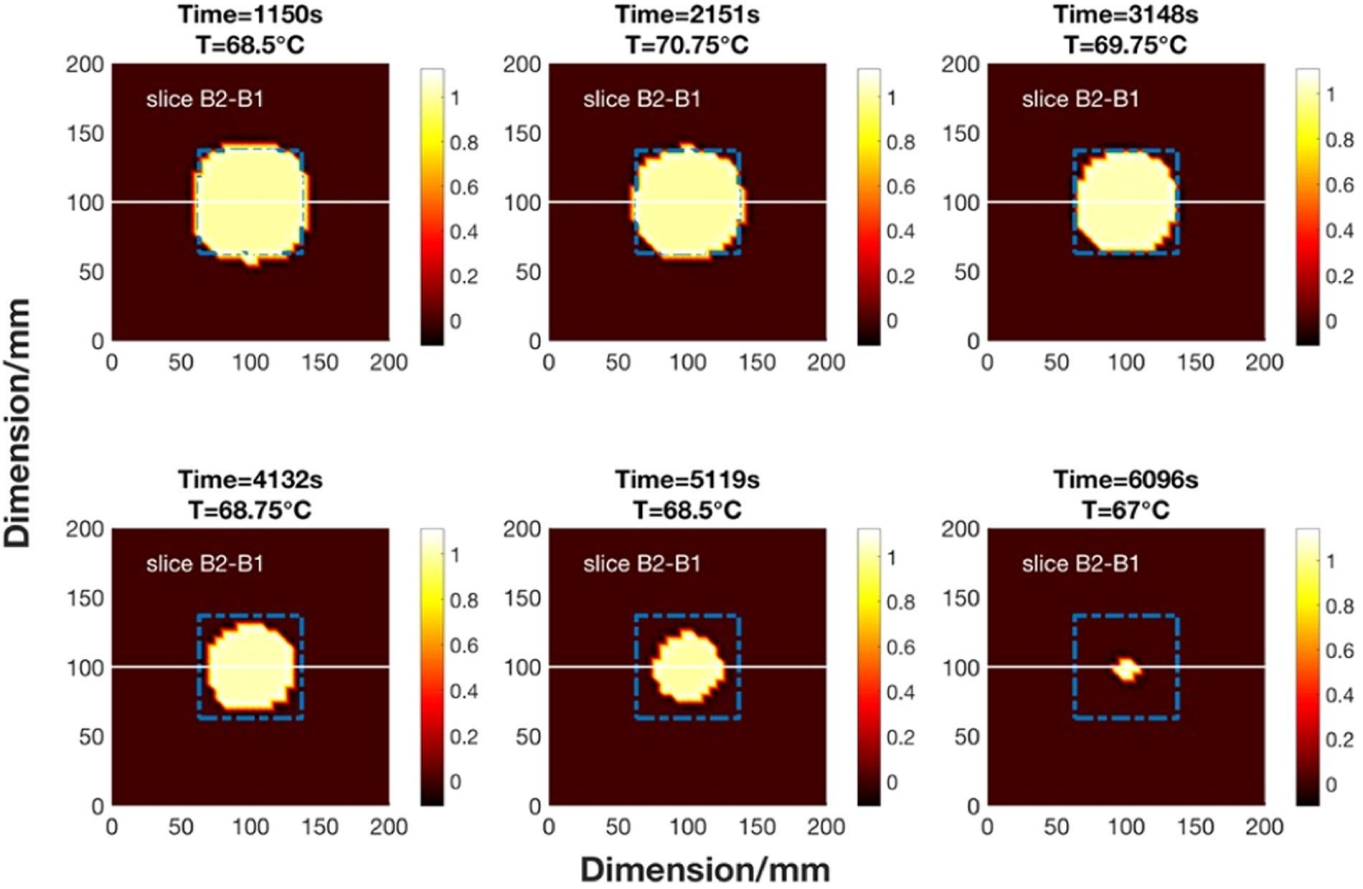
Reconstructed temporal images of the liquid zone during solidification process.

**Figure 13:**
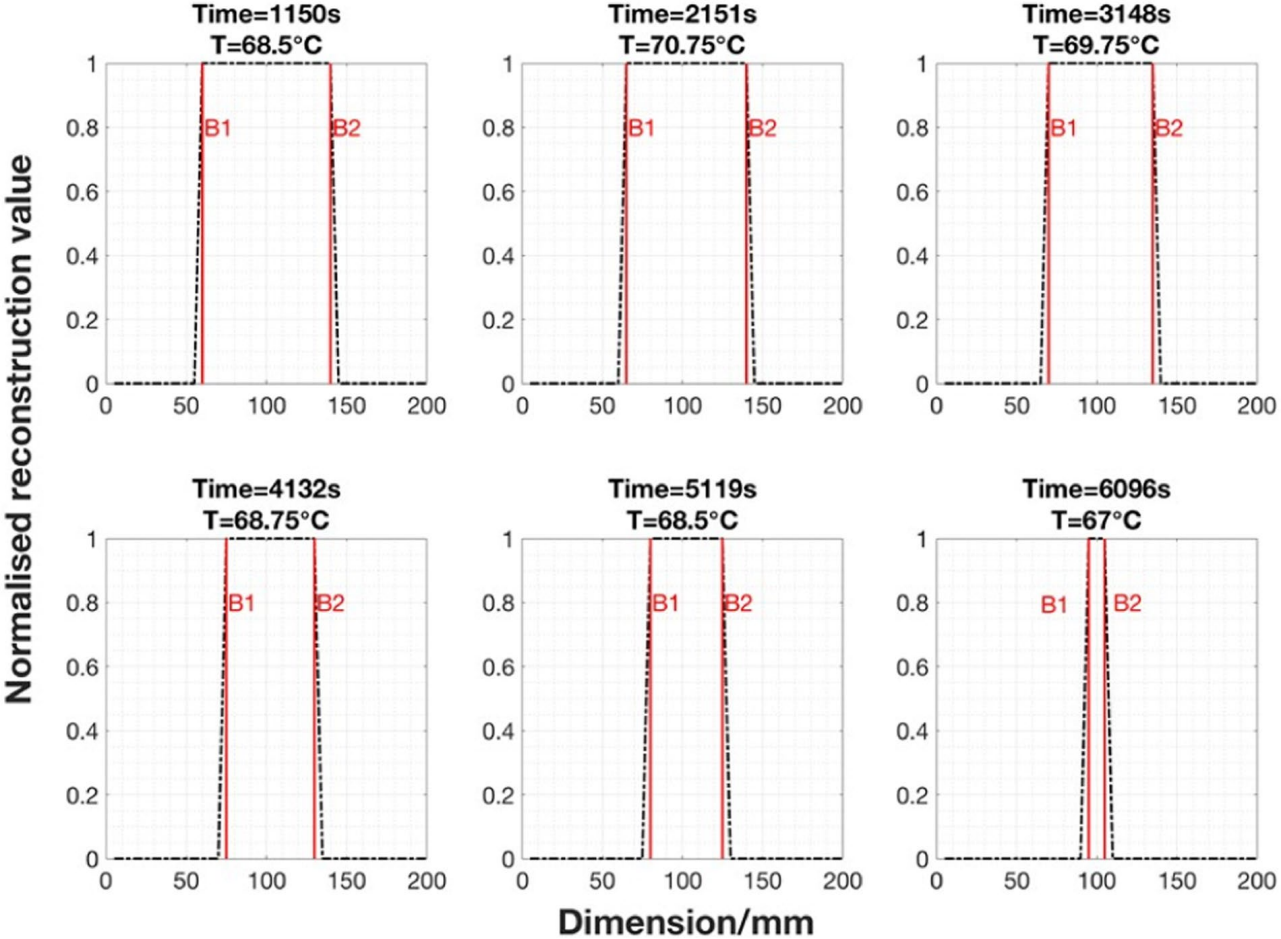
1D cross sectional distribution of the reconstructed temporal images from Fig. 12.

The solidification experiment is also repeated several times using an improved MIT system (Fig. 5). For the experimental results shown below, the experiment takes a little under 3.5 hours. Over this period, a total of 149 frames of *in-situ* MIT data and temperature changes are collected. Noting that the images are reconstructed across the time domain with temporal correlation, not individually. A set of 6 data points are selected for presentation of the results. The image reconstruction and analysis can be described in the following steps. Firstly, each image is reconstructed within a 40-by-40 pixel grid. Figure 11 shows a selection of 1D distributions of the raw reconstructed image values along both x- and y-axis directions. Secondly, a multithresh technique is applied globally to all temporal images to convert the raw reconstructed values to binary values^13^, the selection criteria is such that let $\tilde{\Delta \sigma }=1$ if $\tilde{\Delta \sigma }\ge threshold$ and let $\tilde{\Delta \sigma }\mathrm{=0}$ if $\tilde{\Delta \sigma } < threshold$. The threshold value is a global parameter shown in red in Fig. 11 and the zones above the threshold are the area occupied by a liquid metal in the imaging region. Thirdly, taking the x-cross sectional as an example (due to the proximity of the reconstructed values in x- and y-directions), the boundary of solid and liquid region can be obtained by distinguish between the binary values (Fig. 12). The estimated boundary B1 and B2 are calculated by taking the pixel indices along the x-direction pixels where the first and last 1 values occur within converted binary image grid. Fourthly, the differences between two pixel indices (B2-B1) describe the area of the liquid metal region in grid. Finally, the grid pixel indices can be converted into actual dimension in millimeter and used to estimate the solidified shell thickness (Fig. 13).

**Figure 14:**
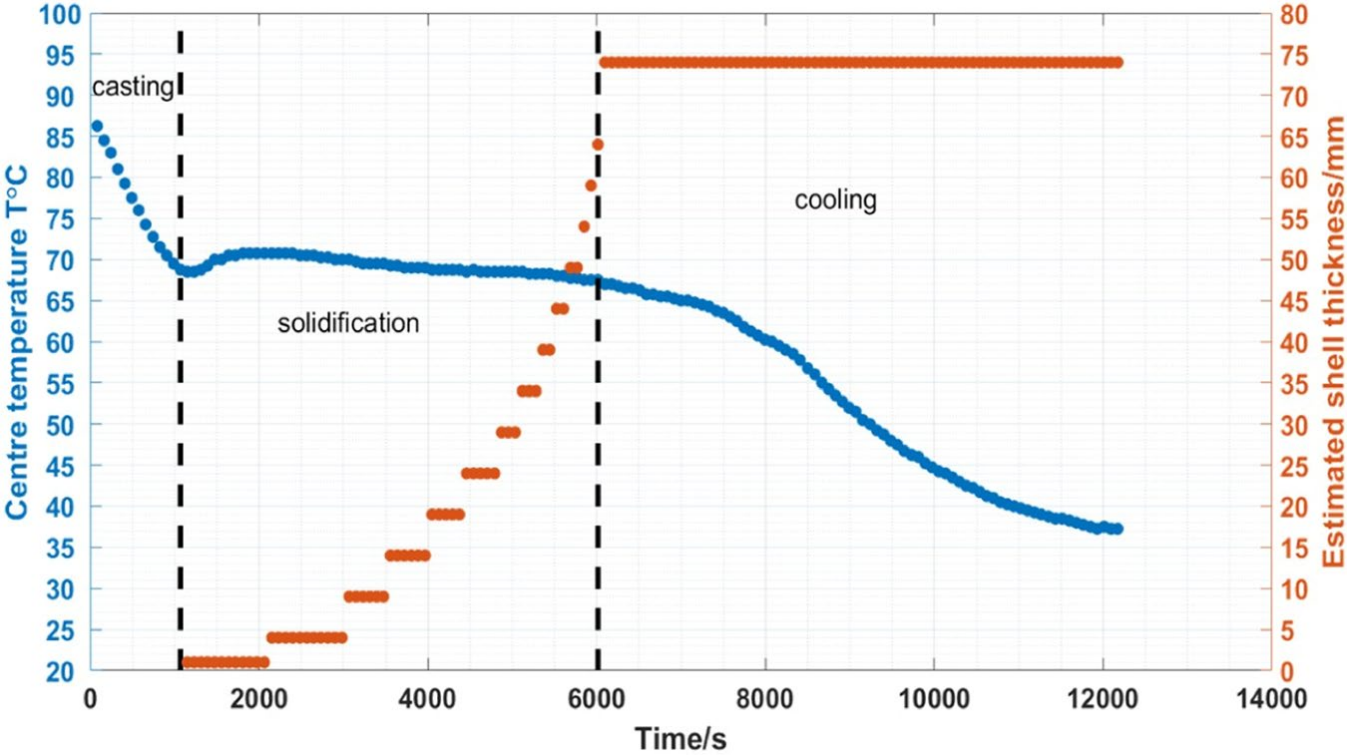
Estimated shell thickness versus the central temperature and time stamp from reconstructed temporal images.

To verify the imaging results, a thin k-type thermocouple is placed in the centre at 60 mm depth of the mould to record the temperature changes continuous during the test. The experiment can be separated into casting, solidification and cooling period. Figure 14 shows the central temperature changes versus time. During the solidification period, the central temperature $T$ initially increases slightly and then deceases steadily. In the cooling period, the central temperature $T$ decrease continuously. The estimated shell thickness versus time is also demonstrated. The casting period is not of interest, as the wood alloy is being poured into the mould. The temporal reconstructed images show some small variations in conductivity during the cooling period, however the temperature measurements suggest that the metal is already fully solidified, consistent with Figs 2 and 4, where the solidification occurs in a relatively narrow temperature window.

## Discussion

A single coil test and an MIT prototype are used to demonstrate the state change of the wood alloy via inductance measurements, relating to the electrical conductivity change. Both Figs 2 and 4 confirm that for eutectic wood alloy, the state change from liquid to solid occur in a narrow temperature window (within a couple of degrees in Celsius). The objective of these two tests is to verify the feasibility of the solidification measurements via electromagnetic induction mechanism and an array of tomographic sensors.

Static cold phantoms tests are carried out to verify the depth detection theory, allowing the proposed MIT system seeing through a metallic shell at a depth of 22 mm. The images are reconstructed individually using a free space measurement as reference. The sizes of the internal metallic inclusions within the outer metallic shells are reliably recovered. It is verified that the differences in phase measurement against a free space measurement have a quadratic relationship with the diameter of the internal rods. This allows the image reconstruction parameters be chosen from the quadratic curve and offers flexibility than a conventional method of taking a reference measurement from an outer metallic enclosure without the presence of an internal rod (which might not be feasible in practice). The cold phantoms tests are set up in an idealised scenario to represent the solid and liquid interface, nevertheless the results demonstrate the system capability and could potentially unlock several new applications that require seeing through metals.

For the real time *in-situ* imaging of the wood alloy solidification, the phase differences are taken against a fully solidified wood alloy as reference measurement. This provides a more steady state transition in the linear approximation. A temporal reconstruction technique is applied to several experimental trials to reveal the solidification process. The results are cross validated against *in-situ* temperature measurements. The proximity in the raw reconstructed values along both x- and y-axis directions in Fig. 11 suggests that the solidification occurs uniformly, dramatic changes are not observed either in temperature nor the position of the liquid metal. It is likely that during industrial continuous casting, the liquid zone might not always be in the central position, the solidification process could also occur at different rates in different directions. Therefore it is anticipated that a 3D imaging method could offer advantage in recovering the axial change in conductivity together with an initial estimation of the distribution of the liquid metal zone from a complete computational fluid dynamics modelling of the process.

This study offers the first impression of the imaging capability of the laboratory prototype. For the deployment of the device in a continuous casting machine, it is suggested that front-end sensors will need to be shielded against high temperatures (approximately 1100–1500 °*C* from liquid metal surface to inner core). The final system could have a similar installation mechanism as commonly seen for an electromagnetic stirrer in a continuous casting application.

Taken together, this paper presents the first real time *in-situ* visualisation and measurements of the solidified shell thickness during molten metal solidification process. The results presented in this paper contribute to the knowledge of solidification process via a magnetic induction tomography technique.
